# The intracellular milieu of Parkinson’s disease patient brain cells modulates alpha-synuclein protein aggregation

**DOI:** 10.1186/s40478-021-01256-w

**Published:** 2021-09-16

**Authors:** Nadja Gustavsson, Ekaterina Savchenko, Oxana Klementieva, Laurent Roybon

**Affiliations:** 1 Medical Microspectroscopy, Department of Experimental Medical Science, Lund University, Lund, Sweden; 2 Stem Cell Laboratory for CNS Disease Modelling, Department of Experimental Medical Science, BMC D10, Lund University, Lund, Sweden

**Keywords:** Parkinson’s disease, Protein aggregation, Alpha-synuclein, Cellular environment, Human iPSCs, Midbrain spheroids

## Abstract

Recent studies suggest that brain cell type specific intracellular environments may play important roles in the generation of structurally different protein aggregates that define neurodegenerative diseases. Using human induced pluripotent stem cells (hiPSC) and biochemical and vibrational spectroscopy techniques, we studied whether Parkinson’s disease (PD) patient genomes could modulate alpha-synuclein (aSYN) protein aggregates formation. We found increased β-sheets and aggregated aSYN in PD patient hiPSC-derived midbrain cells, compared to controls. Importantly, we discovered that aSYN protein aggregation is modulated by patient brain cells’ intracellular milieus at the primary nucleation phase. Additionally, we found changes in the formation of aSYN fibrils when employing cellular extracts from familial PD compared to idiopathic PD, in a Thioflavin T-based fluorescence assay. The data suggest that changes in cellular milieu induced by patient genomes trigger structural changes of aSYN potentially leading to the formation of strains having different structures, properties and seeding propensities.

## Introduction

Protein aggregation is one of the major cellular hallmarks of neurodegenerative diseases (ND). Parkinson’s disease (PD), dementia with Lewy bodies and multiple system atrophy define alpha-synucleinopathies, a group of ND characterized by intracellular aggregation of alpha-synuclein (aSYN) protein [1]. Recent studies suggest that the intracellular environment of specific brain cells (neurons and oligodendrocytes) [2] and post-translational modifications [3], may play important roles in the generation of structurally different protein strains that define ND. However, there is no data that indicates if and how patient-specific brain cells’ intracellular milieus modulate protein aggregation.

Here, we studied changes in intracellular environment of PD patients’ brain cells and how they modulate aSYN protein aggregation, using human induced pluripotent stem cells (hiPSC) and biochemical methods combined with infrared spectroscopy technique. Notably, we performed a Thioflavin T (ThT)-based fluorescence assay to examine the effect of patient genome-induced changes in brain cells intracellular milieu on aggregation of recombinant p.A53T aSYN monomers.

## Case presentation

**Fig. 1:**
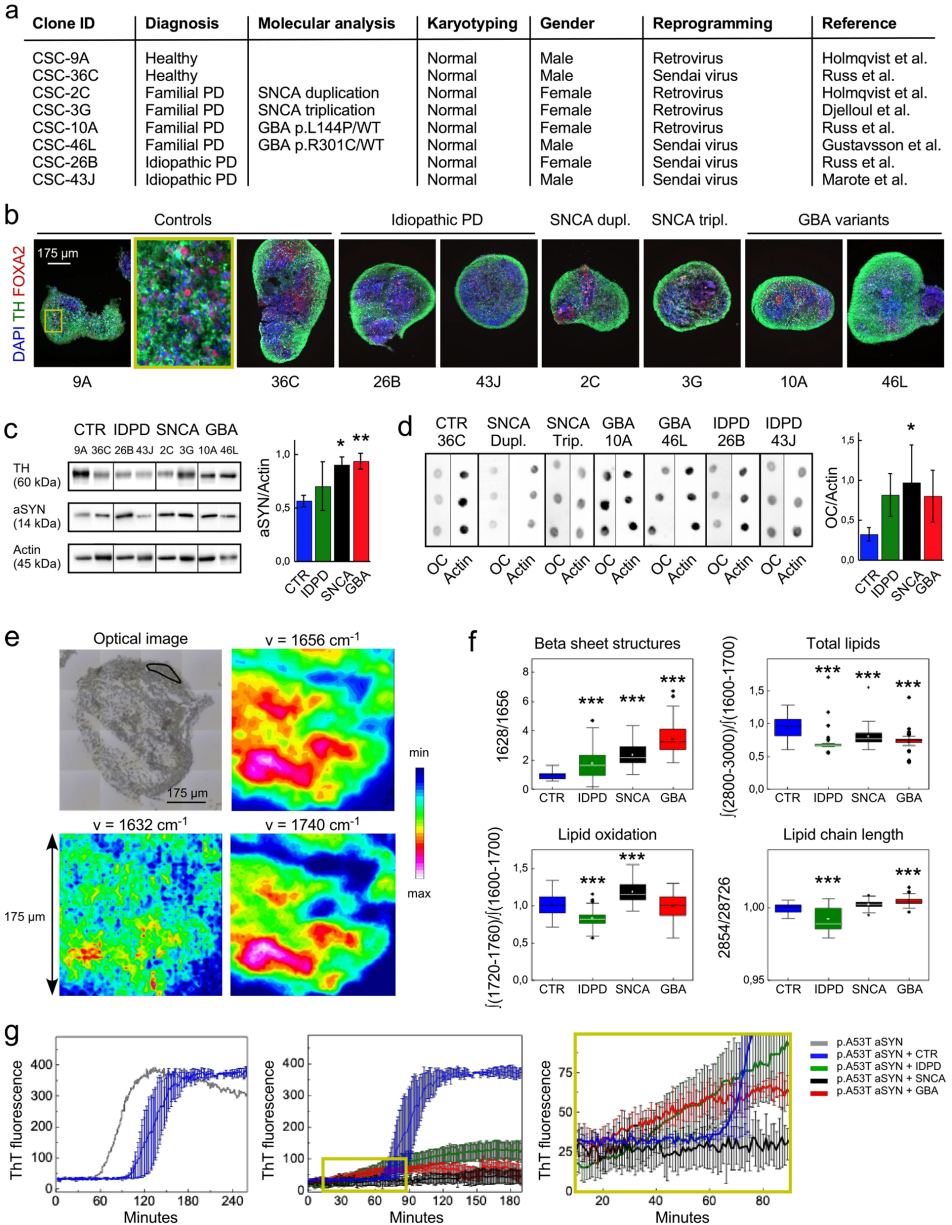
**a** Table summarizing the hiPSC lines employed in the study. **b** Immunohistochemistry performed on 75-day old hiPSC-derived midbrain spheroids revealed abundance of TH/FOXA2-positive dopaminergic neurons. **c** Western blot analysis of RIPA extracts revealed an increase of 14 kDa aSYN level in the SNCA and GBA spheroids. Western blot analysis was performed using antibody specific to aSYN. Data were normalized to the level of actin and presented as mean ± SD; N = 2 patients; one line per patient; *p < 0.05. Statistical analysis was performed by comparing control, SNCA and GBA using one-way ANOVA followed by Tukey’s post hoc test; **p < 0.01 and *p < 0.05. **d** Dot blot analysis revealed increase of aSYN fibrillar content. Dot blot analysis was performed using OC antibody specific to amyloid fibrils. Data are normalized to the control and presented as mean ± SD; N = 2 patients; one line per patient; *p < 0.05. Statistical analysis was performed using a two-tailed t-test. **e** µFTIR analysis further revealed changes in molecular structures in the spheroids models of synucleinopathies. Bright field image of spheroid deposited on CaF_2_. Black line indicates the area of µFTIR analysis. Red-green–blue maps are infrared images at specific frequencies used for structural analysis. **f** Box diagrams show µFTIR analysis of β-sheet structural content, total lipids measured as area ratio of lipids between 2800–3000 cm^−1^ and amide I (1600–1700 cm^−1^); lipid oxidation measured as an area ratio of peaks centered at 1740 cm^−1^ and amide I (1600–1700 cm^−1^); and lipid chain length measured as a ratio between peaks at 2854 cm^−1^ and 2874 cm^−1^, within healthy, *SNCA*^multiplication^, Idiopathic PD and GBA midbrain spheroids. Box diagrams show mean (represented by squares), median line, interquartile range from lowest to highest, and outliers (shown as lozenges). Statistical analysis was performed by comparing all groups using one-way ANOVA followed by Bonferroni post hoc test; ***p < 0.001. **g** PD midbrain spheroids cellular milieus trigger aggregation of aSYN by stimulating primary nucleation of aSYN. The kinetics of 10 μM recombinant p.A53T aSYN aggregation alone and in the presence of extracts from midbrain spheroids (50 ng/μL) was monitored by ThT fluorescence in 10 mM MES pH 5.5 buffer under quiescent conditions at 37 °C. Results are presented as mean ± SD, N = 2 patients; one line per patient

Parkinson's disease patient brain tissue is only available post-mortem when protein aggregation is advanced and adaptive processes are at play [4, 5]. To examine early changes in aSYN protein levels and aggregation, we generated midbrain spheroids from PD patients and healthy controls’ hiPSC using a well-established methodology [6] (Fig. 1a, b). Patient iPSC-derived spheroids are the closest models to human brain parenchyma [7]. Midbrain regionalized spheroids are composed in majority of astroglia positive for glial fibrillary acidic protein (GFAP) and dopaminergic neurons co-expressing the enzyme tyrosine hydroxylase (TH) and the transcription factor FOXA2 (Fig. 1b) [6, 8]. Western blot analysis revealed significant increased levels of total aSYN in PD midbrain spheroids generated from glucocerebrosidase (*GBA*) gene variants and synuclein alpha (*SNCA*) locus multiplication cases, compared to controls (Fig. 1c). Dot blot analysis using OC antibody specifically recognizing fibrillar forms of amyloid [9, 10] and Fourier transform infrared microspectroscopy (µFTIR) measurements revealed increased fibrillar aSYN content and β-sheets in PD midbrain spheroids compared to controls, respectively (Fig. 1c, d).

It has been shown that changes in lipid composition present in familial glucocerebrosidase (GBA) variant forms of PD correlate with changes in aSYN aggregation [11]. Moreover, recent work suggests that changes in lipid oxidation measured by µFTIR correlate with protein aggregation in cellular models of Alzheimer’s disease [12]. µFTIR analysis of the midbrain spheroids allowed us to further evaluate changes in lipid composition, oxidation, and lipid chain length. We found that while the amount of cellular lipids was decreased in PD midbrain spheroids, variation in their oxidation and chain length was dependent on the cells’ genome (Fig. 1e, f). These data suggested that changes in lipids may fluctuate in different forms of PD, which prompted us to examine the effect of intracellular milieu on aSYN protein aggregation.

We examined aggregation of recombinant p.A53T aSYN protein monomers in intracellular environment using a ThT-based fluorescence assay. Interestingly, we found decreased ThT fluorescence for samples containing PD hiPSC-derived midbrain spheroids extracts, compared to controls (Fig. 1g). Notably, there was a lower level of ThT-based fluorescence when employing extracts from familial PD hiPSC-derived midbrain spheroids compared to idiopathic PD ones (Fig. 1g). This finding was counterintuitive since previous work showed acceleration in the reaction of recombinant aSYN fibril formation after the addition of purified lipid extracts that are most abundant in PD *GBA* variant cells [11] or aSYN seeds generated using protein misfolding cyclic amplification [13].

## Discussion and conclusions

Our work is the first to demonstrate that changes in intracellular environment induced by patient genomes are important for aSYN aggregation. Indeed, the cellular lysate of PD patient iPSC-derived brain cells, which also includes membrane components, was capable to modulate aggregation of recombinant p.A53T aSYN monomers. ThT fluorescence was low when aSYN aggregation was allowed to form in the presence of PD extracts. This result was unexpected because increased ThT fluorescence peaks more rapidly when employing two-component systems [11, 13]. We hypothesize that protein interactors present in the PD intracellular milieu reduce ThT binding sites availability [14] or alter the amyloid structure of the formed fibrils. Alternatively, the protein interactors could potentiate the formation of small oligomers having great seeding propensities.

Given the fact that iPSCs have properties similar to embryonic stem cells [15], and that through differentiation they give rise to brain cell types with characteristics of young cells [16], we propose that our data are akin to mechanisms of PD likely occurring early in the life of the patient. The data obtained using iPSCs further suggest that the pathological changes identified in idiopathic PD cases may have a (epi)genetic pathological process underlying.

Future efforts should focus on defining what cellular components modulate the structure and properties of aSYN protein aggregates. Few studies have identified aSYN binding partners [17, 18]; however, it remains to be determined which ones actively contribute to the structure and properties of the aSYN assemblies generated early in life in patient brain cells. Examining similarities and differences in structures and properties of aSYN fibrillar strains that form in different familial forms of PD [*GBA*, *SNCA* and other gene variants], idiopathic PD, and other synucleinopathies [19], would allow enhanced stratification of patients towards personalized treatment(s). Ultimately, therapeutic strategies should focus into restoring normal cellular networks and pathways altered in the disease that trigger the formation of toxic aSYN assemblies. We further propose µFTIR and ThT-based fluorescence assays as diagnostic tools to confirm PD pathology, monitor therapeutic responses, and to measure protein aggregation in the intracellular milieu of hiPSC-derived midbrain spheroids.

## Methods

### Human induced pluripotent stem cell lines

The generation of hiPSCs lines CSC-9A, CSC-36C, CSC2-C, CSC-3G, CSC10A, CSC46L, CSC26B and CSC43J has been previously reported [17, 20–23].

### Generation of midbrain spheroids containing FOXA2+/TH+ neurons

To differentiate the hiPSCs into midbrain spheroids containing dopaminergic neurons, a previously established protocol was employed [6]. Briefly, hiPSCs were detached from feeders with dispase II and seeded in ultra-low attachment flasks in WiCell media supplemented with 10 μM of ROCK inhibitor Y27632 and 20 ng/mL of FGF2 (Thermo Fisher Scientific). The following day, the medium was changed to neural induction medium (NIM) composed of advanced DMEM/F12, 2 mM L-glutamine, 1% NEAA, 1% N2 supplement, 1% P/S (Thermo Fisher Scientific), and supplemented with 0.1 μM LDN, 10 μM SB431542, 200 ng/mL SHH-C, 1 μM SAG and 0.8 μM CHIR (Sigma-Aldrich, SML1046). The medium was replaced every other day. On day 6, SB and SHH-C (Thermo Fisher Scientific, PMC8031) were removed from the medium and SAG concentration was increased to 2 μM; on day 10, LDN was removed. From day 12 onwards, the cells were grown in NIM supplemented with 100 ng/mL FGF8 (Thermo Fisher Scientific, PHG0184), 2 μM SAG, 10 ng/mL BDNF (Peprotech, 450-02-250) and 200 μM ascorbic acid (AA) (Sigma-Aldrich, A4403). From day 22, the medium was replaced with neural differentiation medium (NDM) containing Neurobasal media with 2 mM L-glutamine, 1% NEAA, 1% N2 supplement, 1% B27 without vitamin A, 1% P/S, supplemented with 100 ng/mL FGF8 and 2 μM SAG, 10 ng/mL BDNF, 10 ng/mL GDNF (R&D systems, 212-GD/CF), 200 μM AA (Sigma-Aldrich, A4403), 500 μM db-cAMP (Sigma-Aldrich, D0627) and 1 ng/mL TGF (Peprotech, 100-36E). From day 30 onwards, FGF8 and SAG were removed from the medium and 50 μM DA (Sigma-Aldrich, H850) was added to promote pigmentation of the spheroids.

### Immunostaining and image acquisition

For immunohistochemistry, spheroids were fixed in 4% paraformaldehyde (PFA) and snap-frozen in liquid nitrogen prior to be sectioned into 20 μm thick sections using a cryostat (Leica Microsystems GmH, Wetzlar Germany). Samples were then stored at − 80 °C for immunohistochemical staining. Frozen sections were air-dried for 1 h, then blocked for 1 h at RT in PBS containing 10% donkey serum and 0.1% PBS-Tween 20. Primary antibodies—goat anti-FOXA2 (Santa-Cruz, sc6554, 1:250) and mouse anti-TH (Millipore, MAB318, 1:500), were diluted in blocking solution and sections were incubated overnight at 4 °C. Sections were rinsed and incubated with appropriate Alexa-fluor 488- and 555-conjugated secondary antibodies in PBS (1:400, Thermo Fisher Scientific), for 1 h at room temperature (RT) in the dark. Additionally, cell nuclei were stained with DAPI (1:10,000; Sigma-Aldrich). All fluorescent images were acquired using an LRI—Olympus IX-73 epifluorescence microscope.

### Western and dot blot analysis

Spheroids aged 75 days in vitro (DIV) were homogenized in RIPA buffer (ThermoFisher Scientific, Sweden) complemented with Halt Protease Inhibitor and Halt Phosphatase Inhibitor Cocktails (ThermoFisher Scientific, Sweden). After a short centrifugation step to remove cell debris, the supernatant was collected, and protein concentrations estimated using the BCA protein assay kit (ThermoFisher Scientific, Sweden, #23225). For Western blot, samples were heated at 70 °C for 10 min in tricine SDS sample buffer 2× Novex (#LC1676). Samples were loaded onto 10–20% Tricine SDS-PAGE gels (Sigma-Aldrich, Sweden) and run at 125 V in Tricine SDS Running Buffer (ThermoFisher Scientific, Sweden, #LC167523225). Gels were then fixed in 20% ethanol for 5 min, followed by protein transfer to polyvinylidene difluoride (PVDF) membranes (Sigma-Aldrich, Sweden, #IB24002). Membranes were incubated in 0.4% PFA for 20 min at RT and washed in PBS-T. Membranes were blocked using a 5% skim milk (Sigma-Aldrich) in PBS-Tween 20 (PBST) solution, and incubated overnight with primary antibodies diluted in blocking solution, at 4 °C. Primary antibodies: mouse anti-actin (Sigma-Aldrich, A5441, 1:5000); aSYN (BioLegend, #848301, 1:1000), and TH (Millipore, MAB318, 1:1000). Dot blot samples were deposited directly onto PVDF membranes with OC antibody (Sigma-Aldrich, Sweden, #AB 2286, 1:5000), that targets amyloid fibrils. The blots were incubated with primary antibodies overnight. Next day, membranes were washed and incubated with HRP-conjugated secondary antibodies (1:1000; R&D Systems) for 1 h. Blots were revealed using Pierce Enhanced Chemo-Luminescence solution and imaged using The Ultimate Western Blot Imaging System Azure c600 (AH diagnostics AB, Sweden). Quantification was performed using ImageJ.

### Fourier Transform Infrared microspectroscopy and data analysis

µFTIR analyses were performed for spheroids aged 75 DIV. Briefly, 16 μm cryosectioned spheroid slices were mounted on 1 mm thick CaF_2_ spectrophotometric windows (Eksma Optics, Lithuania) and dried under nitrogen flow. For reproducibility, infrared spectra were taken from areas of the spheroid with maximum TH expression. Spectra were recorded at RT using a Hyperion 3000 IR microscope (Bruker Scientific Instruments, Billerica, MA, USA) coupled to a Tensor 27 with MCT (mercury cadmium telluride) detector. The measuring was performed using a range of 900–4000 cm^−1^, with a 4 cm^−1^ resolution, using 500 co-added scans. The background spectra were collected from a clean area of the same CaF_2_ window. OPUS software (Bruker) was used for atmospheric compensation; Orange software (University of Ljubljana [24]) was used to analyze FTIR spectra. To unmask the band positions and to eliminate the baseline contribution, we used second order derivation of the spectra using Savitsky-Golay of 3rd polynomial order 7 with 11 smoothing points [25]. For µFTIR analysis, we used IR bands in the Amide I region (1630−1615 cm^−1^), which is ascribed to the β-sheet structures. The 2000−3000 cm^−1^ region is defined by lipids, and the 1720−1760 cm^−1^ by lipid oxidation [26–29]. For lipidation, we used the ratio between peak areas of 2800–3000 cm^−1^ and Amide I (1600–1700 cm^−1^), for lipid oxidation, we used the ratio between the peak centered around 1740 cm^−1^ and Amide I (1600–1700 cm^−1^), and for quantification of β-sheet structural content we used IR intensity at 1628 cm^−1^, normalized to total proteins to Amide I max at 1656 cm^−1^.

### Thioflavin T-based fluorescence assay

Recombinant aSYN was produced as previously described [30]. To follow the fibrillation process, 10 μM aSYN was added to 96-well non-binding PEGylated plates and supplemented with 5 mM ThT in MES buffer with pH 5.5. RIPA extracts were added to the wells to reach a final amount of 5 μg of total proteins per well. The plates were incubated at 37 °C up to 72 h in a plate reader (FluoStar Omega or FluoStar Galaxy, BMG Labtech, Offenburg, Germany) under quiescent conditions (excitation filter 440 nm and emission filter 480 nm).

### Statistical analysis

Statistical analysis was carried out using OriginPro2019 software. The FTIR and Dot Blot data was normally distributed and was calculated as means ± standard deviations (SD). Statistical significance was determined using one-way ANOVA, followed by Bonferroni or Tukey’s post hoc tests.

## Data Availability

The datasets used and/or analysed during the current study available from the corresponding author on reasonable request.

## Abbreviations

ND: Neurodegenerative diseases
PD: Parkinson’s disease
aSYN: Alpha-synuclein
hiPSC: Human induced pluripotent stem cells
ThT: Thioflavin T
µFTIR: Fourier transform infrared microspectroscopy
GBA: Glucocerebrosidase
SNCA: Synuclein alpha
GFAP: Glial fibrillary acidic protein
TH: Tyrosine hydroxylase
